# Editorial: Exploring SARS-CoV-2 inflammatory responses and potential targets for treatment

**DOI:** 10.3389/fcimb.2025.1625563

**Published:** 2025-05-26

**Authors:** Kanchan Bhardwaj, Kuan Rong Chan

**Affiliations:** ^1^ Department of Biotechnology, Manav Rachna International Institute of Research Studies, Faridabad, Haryana, India; ^2^ Regional Centre for Biotechnology, NCR Biotech Science Cluster, Faridabad, Haryana, India; ^3^ Program in Emerging Infectious Diseases, Duke-NUS Medical School, Singapore, Singapore

**Keywords:** COVID-19, SARS-CoV-2, IFIT3, antiviral, dysregulated immune response

As the world transitions from pandemic emergency to endemic coexistence with SARS-CoV-2, it remains important to develop effective antivirals and therapeutics to reduce the burden of severe COVID-19, especially for the elderly and immunocompromised where COVID-19 vaccines may be less effective. Emerging evidences have highlighted that the host’s dysregulated immune response to SARS-CoV-2, particularly the uncontrolled inflammation, leads to severe COVID-19 progression. Severe COVID-19 is marked by elevated levels of proinflammatory cytokines such as IL-6, TNF-α, IL-1β, and CXCL10, alongside a paradoxical impairment of interferon (IFN) production. Despite high viral loads, patients often exhibit a suppression of interferon-stimulated genes (ISGs), suggesting a highly evolved viral mechanism for immune evasion or modulation. Multiple SARS-CoV-2 proteins, including Nsp1, Nsp13, ORF3b, ORF6, and ORF8 have been shown to antagonize the host IFN response. Complicating the disease landscape further are presence of comorbidities (e.g., diabetes, renal dysfunction, cardiovascular disease) and the continuous emergence of viral variants.

This Research Topic gathered a series of reviews and original articles that bring out attention to the complex mechanisms involved in the host response to SARS-CoV-2 infection and offer insights for disease management and therapeutic approaches.

Guarienti et al. review the recent research and clinical observations providing a comprehensive overview of the complex pathophysiology of the SARS-CoV-2 infection of multiple organs and the key mechanisms affecting various system including the respiratory system, cardiovascular system, hepatic system, digestive system, renal system, reproductive system and the neurological system. Lee et al. contribute to this discourse by identifying cytokines and soluble immune checkpoint regulators as potential predictors of survival in SARS-CoV-2-infected individuals. These findings highlight the promise of immune profiling for risk stratification and clinical management, especially in identifying patients at risk of severe disease or death.

Variant-driven immunity is addressed by Li et al., who examine immune responses to reinfection with Omicron BA.5 in individuals previously exposed to ancestral or other SARS-CoV-2 variants. Their work suggests that immunological memory and response profiles are variant-dependent and may influence reinfection dynamics and vaccine efficacy. Complementing this, Culebras et al. assessed the immune response elicited by different vaccination regimens in individuals with a prior history of infection or no infection. This work re-emphasizes the view of achieving a robust humoral and cellular immune response through heterologous vaccination.

Another angle is provided by de Castro et al., who report elevated levels of interferon-induced protein with tetratricopeptide repeats 3 (IFIT3) in individuals with exposure to SARS-CoV-2 but remain uninfected or are asymptomatic. Their findings propose IFIT3 as a potential protective factor and add to the growing list of interferon-stimulated effectors that could be targeted therapeutically.

Finally, Kumar et al. review the research in the development of antivirals against SARS-CoV-2, since the emergence of the virus. Such a compilation will be a useful resource for further studies in this direction.

This body of work supports a paradigm shift toward precision immunology in COVID-19 care. It advocates not only for antiviral interventions but also for immunomodulatory strategies that can correct dysfunctional host responses without exacerbating immune suppression. It also reflects the emergence of immune biomarkers as tools for patient stratification.

As future research unfolds, emphasis should be placed on longitudinal patient studies and ongoing surveillance to monitor the evolution of SARS-CoV-2 genome. As genomic mutations accumulate, virus may display altered behavior in ways that impact transmissibility, immune escape, and therapeutic effectiveness. A systematic monitoring of the viral genome evolution, combined with functional studies will not only help us stay ahead of potential surges but also inform future vaccine design and antiviral development.

